# Two new species of *Perenniporia* (Polyporales, Basidiomycota)

**DOI:** 10.3897/mycokeys.69.51652

**Published:** 2020-07-10

**Authors:** Chao-Ge Wang, Shi-Liang Liu, Fang Wu

**Affiliations:** 1 School of Ecology and Nature Conservation, Beijing Forestry University, Beijing 100083, China Beijing Forestry University Beijing China; 2 State Key Laboratory of Mycology, Institute of Microbiology, Chinese Academy of Sciences, Beijing 100101, China Institute of Microbiology Beijing China

**Keywords:** phylogeny, polypore, taxonomy, wood-decaying fungi

## Abstract

Two new species of *Perenniporia*, *P. pseudotephropora***sp. nov.** and *P. subcorticola***sp. nov.**, are introduced respectively from Brazil and China based on morphological characteristics and molecular data. *Perenniporia pseudotephropora* is characterised by perennial, pileate basidiocarps with distinctly stratified tubes, grey pores, tissues becoming dark in KOH, a dimitic hyphal system with slightly dextrinoid arboriform skeletal hyphae and broadly ellipsoid to subglobose, truncate, weakly dextrinoid, cyanophilous basidiospores, measuring 4.9–5.2 × 4–4.8 μm. *Perenniporia subcorticola* is characterised by resupinate basidiocarps, yellow pores with thick dissepiments, tissues becoming dark in KOH, flexuous skeletal hyphae, ellipsoid, truncate and slightly dextrinoid basidiospores, measuring 4.2–5 × 3.5–4.2 µm. The morphologically-similar species and phylogenetically closely-related species to the two new species are discussed.

## Introduction

*Perenniporia* Murrill (Polyporales, Basidiomycetes) is typified by *Polyporus unitus* Pers. (Decock and Stalpers 2006). Species in the genus are important, not only for the wood-decaying, but also for their potential application in both biomedical engineering and biodegradation (Younes et al. 2007; Dai et al. 2009; Zhao et al. 2013; Si et al. 2016). *Perenniporia* is characterised by mostly perennial, resupinate to pileate basidiocarps, a dimitic to trimitic hyphal system with generative hyphae bearing clamp connections, cyanophilous and variably dextrinoid skeletal hyphae or skeletal-binding hyphae in most species and ellipsoid, to subglobose, truncate or not, thick-walled, variably dextrinoid and cyanophilous basidiospores. All *Perenniporia* species cause a white rot (Ryvarden and Gilbertson 1994; Decock and Ryvarden 1999; Cui et al. 2019).

Extensive studies on the genus have been carried out during the last 20 years showing a high species diversity and nowadays, 120 taxa have been found (e.g. Hattori and Lee 1999; Decock 2001a, b; Decock et al. 2001; Dai et al. 2002; Decock and Stalpers 2006; Cui et al. 2007; Xiong et al. 2008; Cui and Zhao 2012; Zhao and Cui 2012; Zhao et al. 2013; Decock and Ryvarden 2015; Jang et al. 2015; Decock 2016; Viacheslav and Ryvarden 2016; Huang et al. 2017; Ji et al. 2017; Liu et al. 2017; Shen et al. 2018; Cui et al. 2019; Zhao and Ma 2019).

According to the phylogenetic analysis, based on ITS and nuclear ribosomal partial LSU DNA sequences, Robledo et al. (2009) demonstrated the fundamental phylogeny of *Perenniporia* s.l., combined with such characteristics as a diversity of the vegetative hyphae and basidiospores morphology. In their study, *Perenniporia* s.s. and *Perenniporia* s.l. were scattered into distinct clades, which is also supported by different morphological traits. Zhao et al. (2013) divided *Perenniporia* s.l. into seven clades, based on ITS and nLSU DNA phylogenetic inferences, each of these seven clades being distinguished by a specific combination of morphological characteristics that supported recognition at the genus level. Some genera, having similar morphological characteristics to *Perenniporia*, such as *Amylosporia* B.K. Cui et al., *Murinicarpus* B.K. Cui & Y.C. Dai, *Vanderbylia* D.A. Reid, *Truncospora* Pilát and *Hornodermoporus* Teixeira, were also proved to form distinct lineages in DNA-based phylogenetic analyses (Cui et al. 2019). Besides, several new species were proved to belong to *Perenniporia*, based on morphological characteristics and phylogenetic evidence, which improved the understanding of the phylogenetic structure of *Perenniporia* (Jang et al. 2015; Huang et al. 2017; Ji et al. 2017; Liu et al. 2017; Zhao and Ma 2019).

During a study of wood-inhabiting polypore from Brazil and China, two unknown species of *Perenniporia* were distinguished by both morphology and molecular data. In this study, the two species are described and illustrated.

## Materials and methods

### Morphological studies

The studied specimens are deposited in the herbaria of the Institute of Microbiology, Beijing Forestry University (**BJFC**) and Universidade Federal de Pernambuco (**URM**). Morphological descriptions are based on field notes and herbarium specimens. Microscopic analyses follow Zhao and Cui (2013). In the description: KOH = 5% potassium hydroxide, IKI = Melzer’s reagent, IKI– = neither amyloid nor dextrinoid, CB = Cotton Blue, CB+ = cyanophilous in Cotton Blue, CB– = acyanophilous, L = arithmetic average of all spore length, W = arithmetic average of all spore width, Q = L/W ratios, n = number of spores/measured from given number of specimens. Colour terms are cited from Anonymous (1969) and Petersen (1996).

### Molecular studies and phylogenetic analysis

A CTAB rapid plant genome extraction kit-DN14 (Aidlab Biotechnologies Co., Ltd, Beijing) was used to obtain PCR products from dried specimens, according to the manufacturer’s instructions with some modifications (Shen et al. 2019; Sun et al. 2020). Two DNA gene fragments, ITS and nrLSU were amplified using the primer pairs ITS5/ITS4 (White et al. 1990) and LR0R/LR7 (http://www.biology.duke.edu/fungi/mycolab/primers.htm). The PCR procedures for ITS and nLSU followed Zhao et al. (2013) in the phylogenetic analyses. DNA sequencing was performed at Beijing Genomics Institute and the newly-generated sequences were deposited in the GenBank database. Sequences generated for this study were aligned with additional sequences downloaded from GenBank, using BioEdit (Hall 1999) and ClustalX (Thompson et al. 1997).

In the study, nuclear ribosomal RNA genes were used to determine the phylogenetic position of the new species. Sequence alignment was deposited at TreeBase (submission ID 26254). Sequences of *Donkioporia expansa* (Desm.) Kotl. and Pouzar and *Pyrofomes demidoffii* (Lév.) Kotl. and Pouzar, obtained from GenBank, were used as outgroups (Zhao et al. 2013).

Phylogenetic analyses, used in this study, followed the approach of Han et al. (2016) and Zhu et al. (2019). Maximum parsimony (MP) and Maximum Likelihood (ML) analyses were conducted for the datasets of ITS and nLSU sequences. The best-fit evolutionary model was selected by hierarchical likelihood ratio tests (hLRT) and Akaike Information Criterion (AIC) in MrModeltest 2.2 (Nylander 2004) after scoring 24 models of evolution by PAUP* version 4.0b10 (Swofford 2002).

The MP topology and bootstrap values (MP-BS) obtained from 1000 replicates were performed using PAUP* version 4.0b10 (Swofford 2002). All characters were equally weighted and gaps were treated as missing. Trees were inferred using the heuristic search option with TBR branch swapping and 1000 random sequence additions. Max-trees were set to 5,000, branches of zero length were collapsed and all parsimonious trees were saved. Descriptive tree statistics tree length (TL), consistency index (CI), retention index (RI), rescaled consistency index (RC) and homoplasy index (HI) were calculated for each Maximum Parsimonious Tree (MPT) generated. Sequences were also analysed using Maximum Likelihood (ML) with RAxML-HPC2 through the CIPRES Science Gateway (www.phylo.org; Miller et al. 2009). Branch support (BT) for ML analysis was determined by 1000 bootstrap replicates.

Bayesian phylogenetic inference and Bayesian posterior probabilities (BPP) were performed with MrBayes 3.1.2 (Ronquist and Huelsenbeck 2003). Four Markov chains were run for 4,650,000 generations until the split deviation frequency value was less than 0.01 and trees were sampled every 100 generations. The first 25% of the sampled trees were discarded as burn-in and the remaining ones were used to reconstruct a majority rule consensus and calculate Bayesian posterior probabilities (BPP) of the clades.

Branches that received bootstrap support for maximum likelihood (ML), maximum parsimony (MP) and Bayesian posterior probabilities (BPP) ≥ 75% (ML-BS), 75% (MP-BT) and 0.95 (BPP) were considered as significantly supported, respectively.

## Results

### Phylogeny results

**Figure 1.**
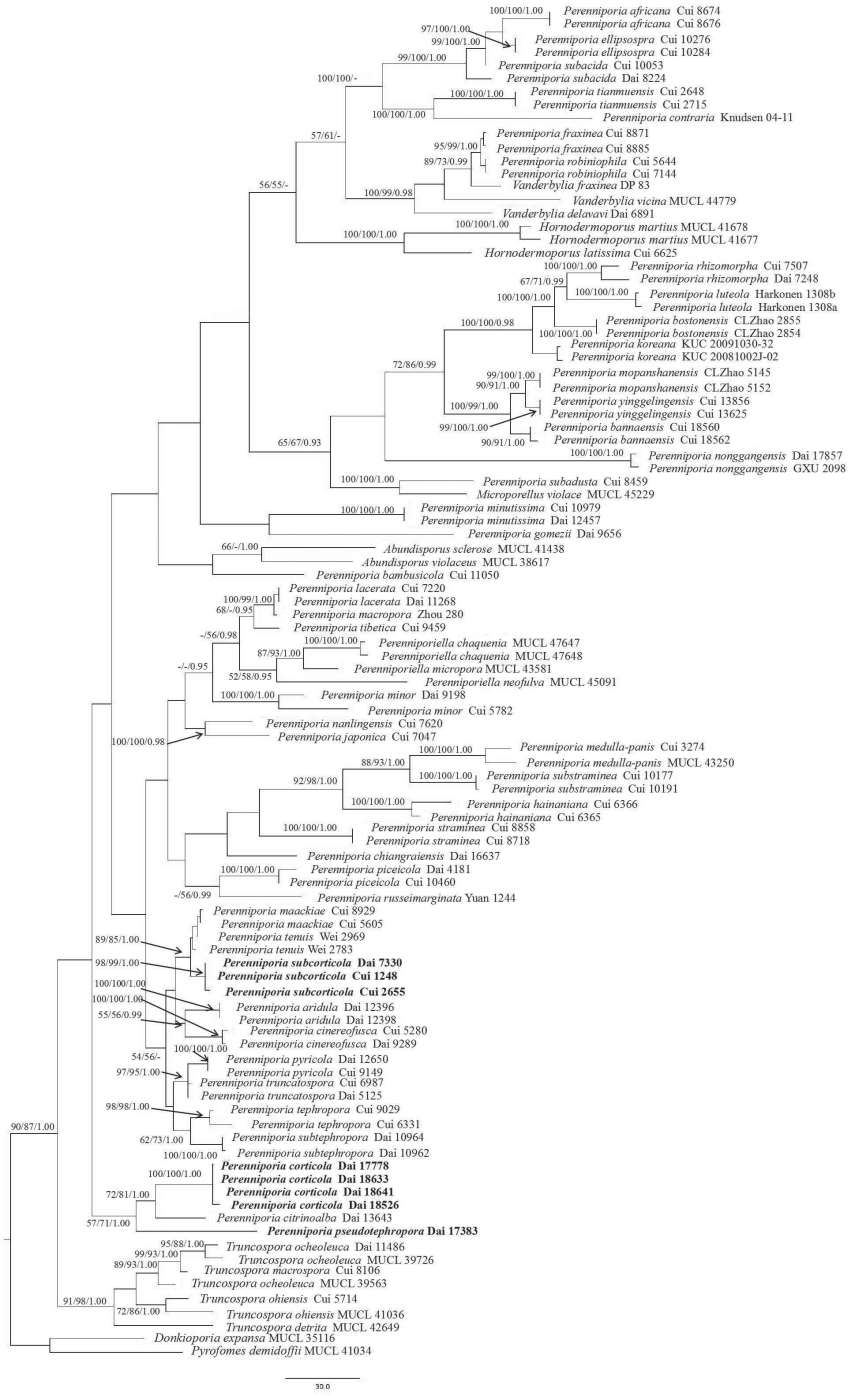
Phylogeny of *Perenniporia* and related species generated by maximum parsimony analysis, based on combined ITS and nLSU sequences. Bootstrap supports for Maximum Likelihood (ML), Maximum parsimony (MP) and Bayesian posterior probabilities (BPP) are not lower than: 50% (ML-BS), 50% (MP-BT) and 0.90 (BPP) on the branches.

The combined ITS and nLSU dataset contained 101 sequences from 101 specimens referring to 59 taxa in this study. They were downloaded from GenBank and the sequences about *Perenniporia corticola*, *P. pseudotephropora* and *P. subcorticola* are new (Table 1). The dataset had an aligned length of 2089 characters in the dataset, of which, 1400 characters are constant, 181 are variable and parsimony-uninformative and 508 are parsimony informative. Maximum Parsimony analysis yielded one equally-parsimonious tree (TL = 2627, CI = 0.389, RI = 0.711, RC = 0.277, HI = 0.611) and a strict consensus tree of these trees is shown in Fig. 1. Best model applied in the Bayesian analysis: GTR+I+G, lset nst = 6, rates = invgamma; prset statefreqpr = dirichlet (1, 1, 1, 1). Bayesian analysis resulted in a same topology with an average standard deviation of split frequencies = 0.009950.

**Table 1.** Information for the sequences used in this study.

Species	Sample namber	ITS	nLSU
*Abundisporus sclerosetosus*	MUCL 41438	FJ411101	FJ393868
*A. violaceus*	MUCL 38617	FJ411100	FJ393867
*Donkioporia expansa*	MUCL 35116	FJ411104	FJ393872
*Hornodermoporus latissima*	Cui 6625	HQ876604	JF706340
*H. martius*	MUCL 41678	FJ411093	FJ393860
	MUCL 41677	FJ411092	FJ393859
*Microporellus violaceo-cinerascens*	MUCL 45229	FJ411106	FJ393874
*Perenniporia africana*	Cui 8674	KF018119	KF018128
*P. africana*	Cui 8676	KF018120	KF018129
*P. aridula*	Dai 12396	JQ001854	JQ001846
	Dai 12398	JQ001855	JQ001847
***P. corticola***	**Dai 17778**	**MT117219**	**MT117224**
	**Dai 18526**	**MT117216**	**MT117221**
	**Dai 18641**	**MT117218**	**MT117223**
	**Dai 18633**	**MT117217**	**MT117222**
*P. bambusicola*	Cui 11050	KX900668	KX900719
*P. bannaensis*	Cui 8560	JQ291727	JQ291729
	Cui 8562	JQ291728	JQ291730
*P. bostonensis*	CL Zhao 2855	MG491285	MG491288
	CL Zhao 2854	MG491284	MG491287
*P. chiangraiensis*	Dai 16637	KY475566	–
*P. cinereofusca*	Dai 9289	KF568893	KF568895
	Cui 5280	KF568892	KF568894
***P. subcorticola***	**Cui 2655**	**HQ654093**	**HQ848483**
	**Dai 7330**	**HQ654094**	**HQ654108**
	**Cui 1248**	**HQ848472**	**HQ848482**
*P. ellipsospora*	Cui 10276	KF018124	KF018132
	Cui 10284	JQ861739	KF018133
*P. fraxinea*	Cui 8871	JF706329	JF706345
*P. fraxinea*	Cui 8885	HQ876611	JF706344
*P. gomezii*	Dai 9656	KX900672	KX900722
*P. hainaniana*	Cui 6366	JQ861745	JQ861761
	Cui 6365	JQ861744	JQ861760
*P. japonica*	Cui 7047	HQ654097	HQ654111
*P. koreana*	KUC 20091030-32	KJ156313	KJ156305
	KUC 20081002J-02	KJ156310	KJ156302
*P. lacerata*	Cui 7220	JX141448	JX141458
	Dai 11268	JX141449	JX141459
*P. luteola*	Harkonen 1308a	JX141456	JX141466
	Harkonen 1308b	JX141457	JX141467
*P. macropora*	Zhou 280	JQ861748	JQ861764
*P. maackiae*	Cui 8929	HQ654102	JF706338
	Cui 5605	JN048760	JN048780
*P. medulla-panis*	MUCL 43250	FJ411087	FJ393875
	Cui 3274	JN112792	JN112793
*P. minor*	Dai 9198	KF495005	KF495016
	Cui 5782	HQ883475	HQ654115
*P. minutissima*	Cui 10979	KF495003	KF495013
	Dai 12457	KF495004	KF495014
*P. mopanshanensis*	CL Zhao 5145	MH784912	MH784916
	CL Zhao 5152	MH784913	MH784917
*P. nanlingensis*	Cui 7620	HQ848477	HQ848486
*P. nonggangensis*	Dai 17857	MT232521	MT232515
	GXU 2098	KT894732	KT894733
*P. piceicola*	Cui 10460	JQ861742	JQ861758
	Dai 4181	JF706328	JF706336
***P. pseudotephropora***	**Dai 17383**	**MT117215**	**MT117220**
*P. pyricola*	Dai 10265	JN048761	JN048781
	Cui 9149	JN048762	JN048782
*P. rhizomorpha*	Dai 7248	JF706330	JF706348
	Cui 7507	HQ654107	HQ654117
*P. robiniophila*	Cui 7144	HQ876608	JF706341
	Cui 5644	HQ876609	JF706342
*P. russeimarginata*	Yuan 1244	JQ861750	JQ861766
*P. straminea*	Cui 8858	HQ654104	JF706334
	Cui 8718	HQ876600	JF706335
*P. subacida*	Cui 10053	KF495006	KF495017
	Dai 8224	HQ876605	JF713024
*P. subadusta*	Cui 8459	HQ876606	HQ654113
*P. substraminea*	Cui 10177	JQ001852	JQ001844
	Cui 10191	JQ001853	JQ001845
*P. subtephropora*	Dai 10964	JQ861753	JQ861769
	Dai 10962	JQ861752	JQ861768
*P. tenuis*	Wei 2969	JQ001859	JQ001849
	Wei 2783	JQ001858	JQ001848
*P. tephropora*	Cui 9029	HQ876601	JF706339
	Cui 6331	HQ848473	HQ848484
*P. tibetica*	Cui 9459	JF706327	JF706333
*P. tianmuensis*	Cui 2648	JX141453	JX141463
	Cui 2715	JX141454	JX141464
*P. truncatospora*	Cui 6987	JN048778	HQ654112
	Dai 5125	HQ654098	HQ848481
*P. yinggelingensis*	Cui 13856	MH427957	MH427965
	Cui 13625	MH427960	MH427967
*Perenniporiella chaquenia*	MUCL 47647	FJ411083	FJ393855
*P. chaquenia*	MUCL 47648	FJ411084	FJ393856
*P. micropora*	MUCL 43581	FJ411086	FJ393858
*P. neofulva*	MUCL 45091	FJ411080	FJ393852
*Pyrofomes demidoffii*	MUCL 41034	FJ411105	FJ393873
*Truncospora detrita*	MUCL 42649	FJ411099	FJ393866
*T. macrospora*	Cui 8106	JX941573	JX941596
*T. ochroleuca*	MUCL 39563	FJ411097	FJ393864
	MUCL 39726	FJ411098	FJ393865
	Dai 11486	HQ654105	JF706349
*T. ohiensis*	MUCL 41036	FJ411096	FJ393863
	Cui 5714	HQ654103	HQ654116
*Vanderbylia delavavi*	Dai 6891	JQ861738	KF495019
*V. fraxinea*	DP 83	AM269789	AM269853
*V. vicina*	MUCL 44779	FJ411095	FJ393862

From the phylogenetic tree (Fig. 1), *P. pseudotephropora* and *P. subcorticola* were absorbed in the genus *Perenniporia*. Moreover, *P. subcorticola* formed a direct lineage with a high approval rating (98/99/1.00) and *P. pseudotephropora* produced an independent lineage.

### Taxonomy

#### Perenniporia pseudotephropora

Taxon classificationFungiPolyporalesPolyporaceae

Chao G. Wang & F. Wu sp. nov.

104E962B-747D-5239-8468-2B5DDAEF5645

MycoBank No: 835122

Figs 2 , 3

##### Diagnosis.

The very thick dissepiments (thicker than pore diameter), tissues becoming pale olivaceous to dark in KOH, flexuous and arboriform skeletal hyphae, ellipsoid to globose, truncate and slightly dextrinoid basidiospores measuring 4.9–5.2 × 4–4.8 μm highlight the species in *Perenniporia*.

##### Holotype.

Brazil. Manaus, Parque Municipal Cachoeira das Orqideas, on rotten angiosperm wood, 12. V. 2017, Y.C. Dai 17383 (BJFC024919).

##### Etymology.

*Pseudotephropora* (Lat.): referring to the species similar to *Perenniporia tephropora*.

##### Basidiocarps.

Perennial, resupinate or effused-reflexed to pileate, without odour or taste when fresh, becoming hard corky when dry. Pilei applanate, semicircular to fan-shaped, projecting up to 1 cm, 3.5 cm wide and about 1 cm thick at base. Pileal surface pinkish-buff, grey to greyish-brown, smooth. Pore surface greyish to pale brown; pores tiny, round, 8–9 per mm; dissepiments thick, thicker than pore diameter, entire. Context thin, fawn to brown, corky, up to 0.5 mm thick. Tubes buff to brown, darker than pore surface, distinctly stratified, hard corky, up to 9.5 mm long.

**Figure 2.**
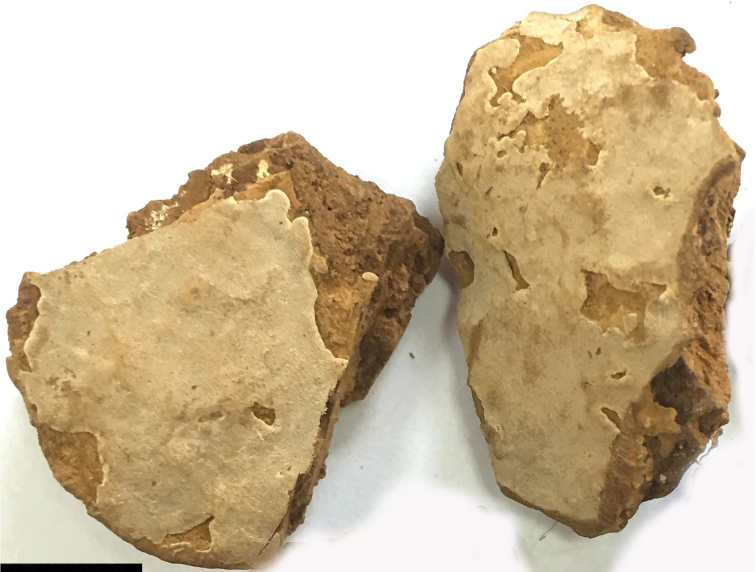
Basidiocarps of *Perenniporia pseudotephropora* (Holotype, Y.C. Dai 17383). Scale bar: 1 cm. Photo by Fang Wu.

##### Hyphal structure.

Hyphal system dimitic; generative hyphae bearing clamp connections; skeletal hyphae arboriform branched, slightly dextrinoid, CB+; tissues becoming pale olivaceous to dark in KOH.

##### Context.

Generative hyphae infrequent, hyaline, thin-walled, bearing clamp connections, 1.6–2.2 μm in diam.; skeletal hyphae dominant, thick-walled with a wide lumen, hyaline to pale brown, frequently arboriform branched, flexuous, interwoven, 1.5–2.8 μm.

**Figure 3.**
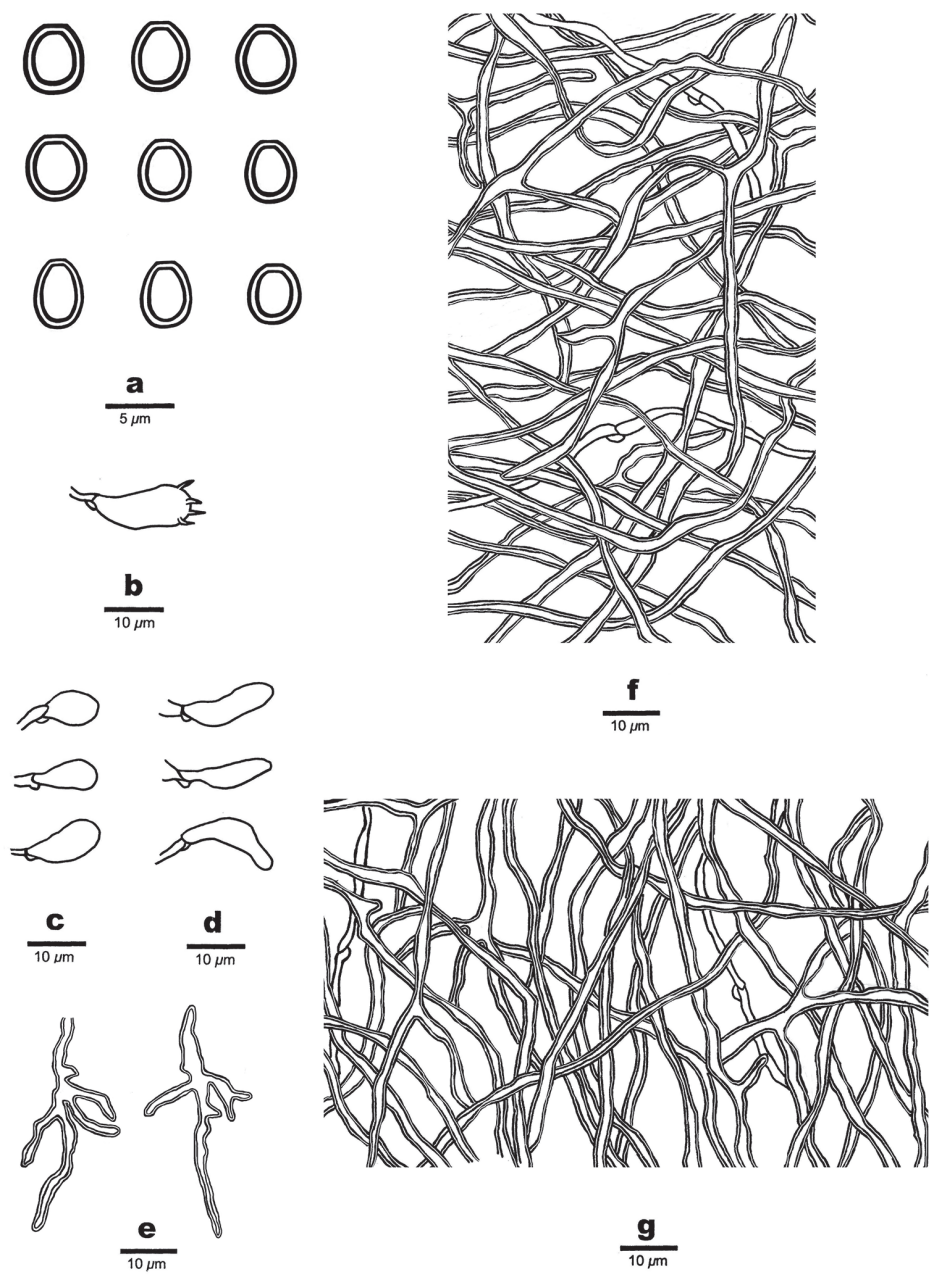
Microscopic structures of *Perenniporia pseudotephropora* (Holotype, Dai17383) **a** basidiospores **b** A basidium **c** basidioles **d** cystidioles **e** arboriform skeletal hyphae **f** hyphae from trama **g** hyphae from context.

##### Tubes.

Generative hyphae infrequent, hyaline, thin-walled, bearing clamp connections, 1.5–2 μm in diam.; skeletal hyphae dominant, thick-walled with a wide lumen, hyaline to pale brown, frequently arboriform branched, flexuous, interwoven, 1.5–3 μm in diam. Cystidia absent, cystidioles present, clavate or fusoid, hyaline, thin-walled, 11–12.5 × 3–4 μm; basidia barrel- to pear-shaped, with four sterigmata and a basal clamp connection, 12.3–13.7 × 6.2–7.5 μm; basidioles in shape similar to basidia, but smaller.

##### Spores.

Basidiospores broadly ellipsoid to subglobose, hyaline to pale brown, truncate, thick-walled, smooth, slightly dextrinoid, CB+, (4.5–)4.9–5.2(–5.3) × 4–4.8(–5) μm, L = 5.02 μm, W = 4.22 μm, Q = 1.19 (n = 30/1).

#### Perenniporia subcorticola

Taxon classificationFungiPolyporalesPolyporaceae

Chao G. Wang & F. Wu sp. nov.

D3D5F733-9792-5064-997F-8512CB06B47D

MycoBank No: 835519

Figs 4 , 5

##### Diagnosis.

*Perenniporia subcorticola* is characterised by resupinate basidiocarps, yellow pores with thick dissepiments, tissues becoming dark in KOH, flexuous skeletal hyphae, ellipsoid, truncate and slightly dextrinoid basidiospores measuring 4.2–5 × 3.5–4.2 µm.

##### Holotype.

China. Fujian Province, Wuyishan Nature Reserve, on rotten wood of *Pinus*, 21.X.2005, Y.C. Dai 7330 (BJFC001421).

##### Etymology.

*Subcorticola* (Lat.): referring to the species similar to *Perenniporia corticola*.

**Figure 4.**
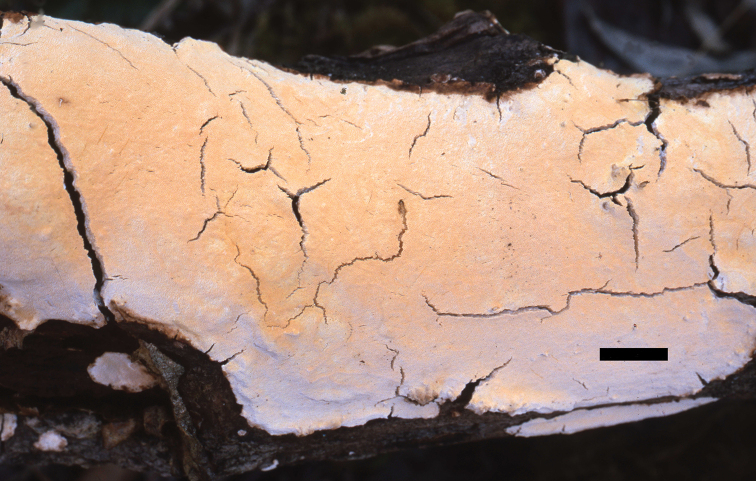
A basidiocarp of *Perenniporia subcorticola* (from Dai 3257). Scale bar: 1 cm. Photo by Yu-Cheng Dai.

##### Basidiocarps.

Perennial, resupinate, soft corky and without odour or taste when fresh, becoming corky when dry, up to 10 cm long, 5 cm wide, 3.5 mm thick at centre. Pore surface yellow when fresh, becoming buff-yellow to curry-yellow when dry; margin narrow, thinning out; pores tiny, round, 7–8 per mm; dissepiments thick, entire. Subiculum thin, cream, up to 2 mm thick. Tubes concolorous with pore surface, up to 1.5 mm long.

##### Hyphal structure.

Hyphal system dimitic; generative hyphae with clamp connections; skeletal hyphae weakly dextrinoid, CB+; tissues darkening in KOH.

##### Subiculum.

Generative hyphae infrequent, hyaline, thin-walled, occasionally branched, 2–3 µm in diam.; skeletal hyphae dominant, thick-walled with a wide lumen, frequently branched, interwoven, 2–3.5 µm in diam.

##### Tubes.

Generative hyphae infrequent, hyaline, thin-walled, occasionally branched, 2–3µm in diam.; skeletal hyphae dominant, thick-walled with a wide lumen, frequently branched, interwoven, 1.8–3 µm in diam. Cystidia absent, fusoid cystidioles present, hyaline, thin-walled, 14–18 × 4.5–7.5 µm; basidia barrel-shaped, with four sterigmata and a basal clamp connection, 13–16 × 6.5–9 µm; basidioles dominant, mostly pear-shaped to capitate, slightly smaller than basidia.

**Figure 5.**
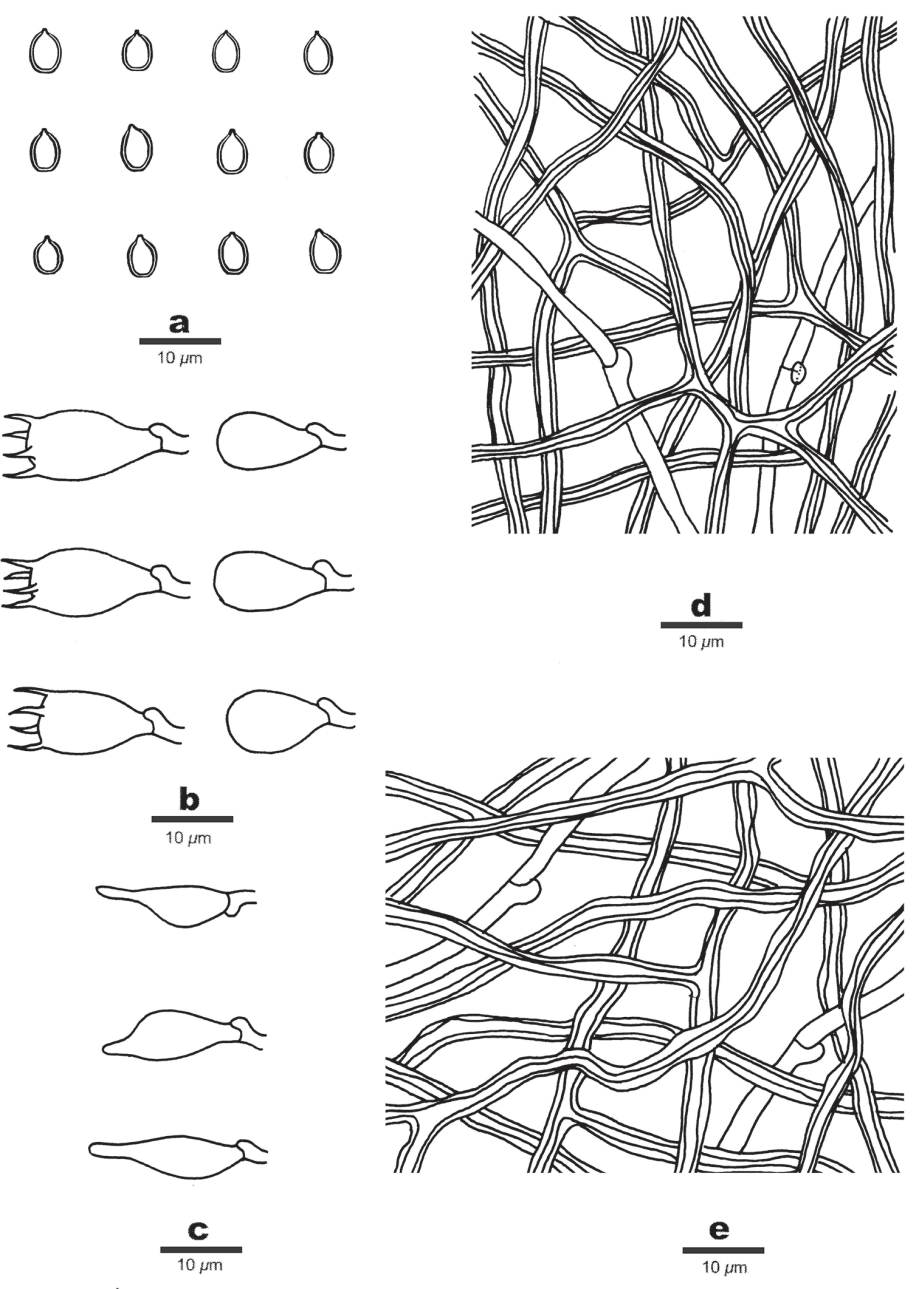
Microscopic structures of *Perenniporia subcorticola* (Holotype, Dai 7330) **a** basidiospores **b** basidia and basidioles **c** cystidioles **d** hyphae from trama **e** hyphae from subiculum.

##### Spores.

Basidiospores ellipsoid, truncate, hyaline, thick-walled, smooth, dextrinoid, CB+, (4–)4.2–5(–5.5) × (3–)3.5–4.2(–4.7) µm, L = 4.66 µm, W = 3.91 µm, Q = 1.16–1.23 (n = 60/2).

##### Additional specimens (paratypes) examined.

China. Hunan Province, Liuyang, Daweishan Forest Park, fallen angiosperm trunk, 21.XII.2000, Dai 3257 (BJFC009205); Zhejiang Province, Tianmushan Nature Reserve, on fallen angiosperm branch, 10.X.2005, Cui 2655 (BJFC001422).

#### Perenniporia corticola

Taxon classificationFungiPolyporalesPolyporaceae

(Corner) Decock, Mycologia 93: 776 (2001)

6504A3BD-FE28-5B3D-A66C-2BB20B357668

Fig. 6

##### Note.

*Perenniporia corticola* and *P. dipterocarpicola* Hattori & S.S. Lee were described from Malaysia (Corner 1989; Hattori and Lee 1999). Decock (2001a) restudied the types of the two taxa and treated *P. dipterocarpicola* as a synonym of *P. corticola*. *Perenniporia corticola* grows on *Dipterocarpus* in lowland forests of Southeast Asia (Decock 2001a; Hattori and Lee 1999) and was not phylogenetically analysed. In this study, *P. corticola* is closely related to *P. citrinoalba* and *P. pseudotephropora.* However, *P. citrinoalba* has larger basidiospores, 5.5–6 ×4.7–5.2 µm (Cui et al. 2019); while basidiospores are 4.6–5(–5.1) × 3.5–4(–4.1) μm in *P. corticola* (4.4–5 × 3.4–4 μm from the type, Decock 2001a). *Perenniporia pseudotephropora* differs from *P. corticola* by resupinate or effused-reflexed to pileate basidiocarps with greyish to pale brown pores, absence of dendrohyphidia and larger basidiospores (4.9–5.2 × 4–4.8 μm vs. 4.6–5 × 3.5–4 μm).

**Figure 6.**
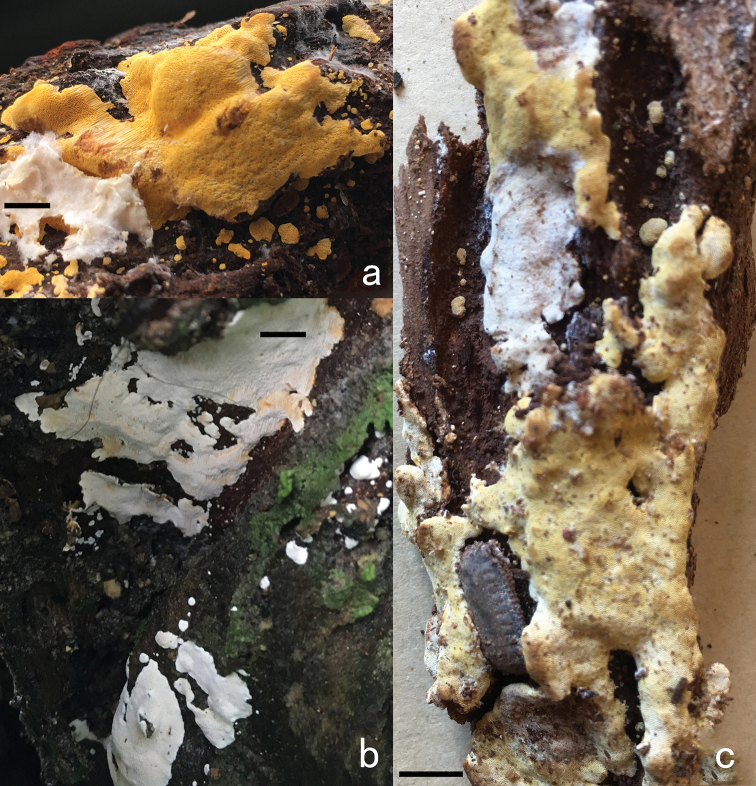
Basidiocarps of *Perenniporia corticola***a** Dai 18641 **b** Dai 18633 **c** Dai 17778. Scale bars: 1 cm. Photos by Yu-Cheng Dai.

##### Specimens examined.

Malaysia. Selangor, Kota Damansara, Community Forest Reserve, on angiosperm stump, 17. IV. 2018, Y.C. Dai 18641 (BJFC026929), Y.C. Dai 18633 (BJFC026921); Taman Botani Negara Shah Alam, on rotten angiosperm wood, 12. IV. 2018, Y.C. Dai 18526 (BJFC026815), Singapore. Singapore Botanical Garden, on rotten angiosperm wood, 17. VII. 2017, Y.C. Dai 17778 (BJFC025310).

## Discussion

*Perenniporia pseudotephropora* is somehow related to *P. corticola* and *P. citrinoalba* B.K. Cui, C.L. Zhao & Y.C. Dai in our phylogeny (Fig. 1). However, the latter two species have completely resupinate basidiocarps with white to yellow pores. *Perenniporia corticola* has smaller basidiospores, 4.6–5 × 3.5–4 μm, while *P. citrinoalba* has larger basidiospores, 5.5–6 × 4.7–5.2 (Cui et al. 2019) vs. 4.9–5.2 × 4–4.8 μm in *P. pseudotephropora*.

*Perenniporia tephropora* (Mont.) Ryvarden is similar to *P. pseudotephropora* in having perennial, resupinate to pileate basidiocarps with grey or greyish to pale brown pore surface, tissues becoming pale olivaceous to dark in KOH and broadly ellipsoid, truncate, dextrinoid basidiospores (Ryvarden and Johansen 1980; Corner 1989) . However, *P. tephropora* has larger pores (4–6 per mm, Ryvarden and Johansen 1980). In addition, the two species are phylogenetically distantly related.

Phylogenetically, *Perenniporia subcorticola* is related to *P. maackiae* (Bondartsev & Ljub.) Parmasto and *P. tenuis* (Schwein.) Ryvarden (Fig. 1) and all these three species have yellow pores. However, *P. maackiae* has effused-reflexed basidiocarps, strongly dextrinoid skeketal hyphae, ellipsoid basidiospores measuring 5–6.5 × 3.5–4.5 μm and grows exclusively on *Maackia* (Dai et al. 2002); while *P. subcorticola* has completely resupinate basidiocarps, weakly dextrinoid skeketal hyphae, basidiospores measuring 4.2–5 × 3.5–4.2 µm and grows on a different tree. *Perenniporia tenuis* is different from *P. subcorticola* by larger pores (3–5 per mm), distinct dextrinoid skeketal hyphae and slightly larger basidiospores measuring 5.5–6.5 × 4.5–5.5 µm (Dai et al. 2002).

Macromorphologically, *Perenniporia subcorticola* is similar to *P. corticola* by its yellow pores and almost the same size of basidiospores and that is the reason why the specimens of *P. subcorticola* were previously treated as P. cf. subcorticola (Dai et al. 2002). However, *P. corticola* has arboriform branched skeletal hyphae and dendrohyphidia at dissepiments and it is a tropical species usually growing on the wood of Dipterocarpaceae (Decock 2001a); while *P. subcorticola* lacks arboriform branched skeletal hyphae and dendrohyphidia and it seems to be a warm temperate species growing on both gymnosperm and angiosperm wood.

*Perenniporia xantha* Decock & Ryvarden and *P. subcorticola* have yellow hymenophore and almost the same size of pores and basidiospores, but *P. xantha* has arboriform skeletal hyphae, lacks cystidioles and its basidiospores are weakly dextrinoid (Decock and Ryvarden 1999); while *P. subcorticola* lacks arboriform skeletal hyphae, has cystidioles and its basidiospores are distinctly dextrinoid.

## Supplementary Material

XML Treatment for Perenniporia pseudotephropora

XML Treatment for Perenniporia subcorticola

XML Treatment for Perenniporia corticola
